# Coffee By-Products Studied by the Planar Ames Bioassay with pH Indicator Endpoint Using the 2LabsToGo-Eco

**DOI:** 10.3390/toxics13090739

**Published:** 2025-08-31

**Authors:** Maryam Monazzah, Cedric Herrmann, Gertrud E. Morlock, Jannika Fuchs, Dirk W. Lachenmeier

**Affiliations:** 1 Chemisches und Veterinäruntersuchungsamt (CVUA) Karlsruhe, Weissenburger Strasse 3, 76187 Karlsruhe, Germany; maryam.monazzah@studenti.unipd.it (M.M.);; 2 Department of Comparative Biomedicine and Food Science, University of Padua, Viale dell’Università 16, 35020 Legnaro, Italy; 3 Institute of Nutritional Science, Chair of Food Science, Justus Liebig University Giessen, Heinrich-Buff-Ring 26-32, 35392 Giessen, Germany; gertrud.morlock@uni-giessen.de

**Keywords:** *Coffea* leaves, *Coffea* blossoms, *Coffea* cherries, *Coffea* silverskin, mutagenicity screening, *Salmonella* bioassay, thin-layer chromatography

## Abstract

The mutagenic potential of coffee by-products, including *Coffea* leaves, blossoms, cherries, and silverskin, was studied using thin-layer chromatography (TLC) coupled with the recent planar Ames bioassay via pH indicator endpoint. The 2LabsToGo-Eco allowed for the separation and detection of mutagens in complex samples. Hot water was the most effective extraction solvent in terms of yield and closely simulated the typical human consumption of coffee by-products. Separation was performed on TLC plates with a mixture of ethyl acetate, *n*-propanol, and water, followed by bioassay detection. The positive control 4-nitroquinoline 1-oxide exhibited clear mutagenic responses, confirming the proper bioassay performance. In the Ames bioautogram, none of the tested coffee by-products showed mutagenic zones, suggesting the absence of strongly acting, acute mutagens under the applied test conditions; however, given the only 5 h short incubation and the use of TA98 strain only, a longer incubation time and testing with additional *Salmonella* strains is recommended. The results provide new safety data for *Coffea* leaves and blossoms and are consistent with some previous studies demonstrating the safety of coffee by-products. However, further improvements in the sensitivity and selectivity of the planar Ames bioassay are demanded, and further in vivo and long-term safety studies are recommended. Considering natural variability, the different uses of pesticides and treatments, and the fluctuating supply chains, coffee by-products may differ highly. The planar bioassay technology using the affordable 2LabsToGo-Eco is a powerful toxicological screening option for the coffee industry, considering the increasing interest in utilizing coffee by-products.

## 1. Introduction

Coffee is one of the most widely consumed beverages globally, with daily consumption exceeding 3.5 billion cups. Beyond its cultural and dietary significance, coffee is a major agricultural product with a substantial impact on international trade and the global economy [1,2]. *Coffea arabica* L. and *Coffea canephora* Pierre ex A. Froehner are the two primary species cultivated for commercial production, both of which are predominantly grown in tropical and subtropical regions [3]. While coffee production traditionally focuses on the utilization of the bean, the whole coffee fruit (cherry) comprises several other components—such as pulp, husk, mucilage, and silverskin—that are often discarded during processing. There has been a growing focus on the valorization of these by-products, also including the leaves and blossoms, due to their promising nutritional, functional, aromatic, and bioactive properties, supporting potential applications in the food, pharmaceutical, and cosmetic industries [4]. Increasing environmental awareness and consumer demand for sustainable solutions have driven significant interest in natural resource valorization and repurposing. Coffee by-products are increasingly viewed as valuable ingredients in green formulations in the food industry due to their biocompatibility and environmental benefits [5]. Within the European Union (EU), the use of such by-products as food requires compliance with the novel food regulation EU No. 2015/2283 [6]. Novel food must undergo a rigorous authorization, including safety assessment based on toxicological data [7].

Mutagenicity testing is crucial for risk assessment of novel foods and ingredients. The European Food Safety Authority (EFSA) recommends a tiered approach, beginning with in vitro tests and advancing to in vivo studies only if needed. The initial testing stage includes standard in vitro assays, such as the Ames test (OECD TG 471) and the in vitro micronucleus assay (OECD TG 487) [8,9]. The Ames test uses *Salmonella* strains that cannot produce histidine and assesses mutagenicity by counting revertant colonies grown on histidine-free culture medium. A positive result indicates that back mutations caused by point mutations, such as base substitutions or frameshifts, restore histidine synthesis [10,11]. The colony-count Ames assay was performed not only in the Petri dish but also by agar overlay on a TLC plate in the 1980s [12]. The Ames microtiter plate format (MPF) is a miniaturized, liquid-based adaptation of the traditional colony-count Ames test, conducted in 384-well plates with a pH indicator endpoint to detect revertants. However, the Ames MPF assay has limitations, such as low sensitivity and matrix interference in complex samples such as food contact materials [13,14]. It is primarily recommended for testing single compounds rather than complex mixtures such as coffee by-products, as the presence of acidic constituents, colored components, or other interfering substances may lead to false-positive or false-negative results, potentially compromising the accuracy of the mutagenicity assessment [15].

Conflicting findings regarding the mutagenicity of coffee have been reported [16]. While some studies using the Ames test have demonstrated mutagenic effects for roasted, instant, and decaffeinated coffee [17,18,19], others have not observed such effects [20,21,22]. Two studies using the traditional Ames test and comet assay with the human hepatocellular carcinoma cell line HepG2 showed no mutagenic/genotoxic effects of spent coffee [23,24], which contradicts another study emphasizing environmental risks from improper waste disposal using the traditional Ames test with specific *Salmonella* Typhimurium strains and the micronucleus assay with murine peripheral cells [25]. These discrepancies may be due to differences in experimental design, methodology, and assay sensitivity. As the mutagenicity of coffee by-products has hardly been investigated, the expanding use of coffee by-products in functional foods (novel foods) and sustainable materials also demands comprehensive safety assessments. Whole coffee fruit extracts from *Coffea arabica* were generally found to be non-mutagenic and non-genotoxic using the micronucleus assay and traditional Ames test with specific *Salmonella* Typhimurium and *Escherichia coli* strains [26]. The comet assay revealed that a coffee silverskin extract was non-cytotoxic and non-genotoxic [27].

Thin-layer chromatography (TLC) or its high-performance HPTLC version is a well-established technique widely used in food analysis and quality control due to its ability to simultaneously analyze complex samples [28]. In contrast to column chromatographic techniques, where analytes are typically eluted and discarded, this system retains compounds on the chromatographic layer and allows for the subsequent in-depth characterization of zones of interest regarding their effects and structures [29,30]. Due to its open-format design, TLC/HPTLC can be coupled with planar bioassays to minimize matrix interference and detect beneficial compounds like antioxidative, anti-diabetes, and anti-Alzheimer substances [31,32] or hazardous compounds including genotoxic, cytotoxic, antibacterial, neurotoxic, and endocrine-disrupting substances [33,34,35], while supporting the Replacement, Reduction, and Refinement (3R) principles in toxicological testing [36]. Planar on-surface bioassays were validated by comparing them with counterpart microtiter plate formats, confirming their reliability [37,38]. Recent developments have expanded the potential of HPTLC by incorporating qualitative image analysis for profiling [39,40] and clustering [41] or quantitative image analysis [42,43] and advanced detection techniques, such as Raman spectroscopy, high-resolution mass spectrometry, and nuclear magnetic resonance spectroscopy, to provide useful structural information on the compounds [37,44,45,46]. These capabilities make hyphenated HPTLC [47,48] particularly valuable for effect-directed analysis, allowing for the fast assignment of bioactive substances in complex mixtures without the need for complex sample preparation [33].

All required equipment in both HPTLC and bioassay laboratories was miniaturized into the all-in-one 2LabsToGo-Eco, allowing for portable on-site analysis with zero-energy consumption (solar panels) and fast, cost-effective, sustainable, and environmentally friendly non-target screening [49,50]. The 2LabsToGo-Eco makes it easy to focus on active or potentially hazardous compounds in complex samples, especially because it can detect their biological or toxicological effects. It yields results comparable in accuracy and reliability to those obtained using conventional, full-scale analytical equipment [33,34,50,51,52,53,54,55].

In this study, after selecting a suitable extractant and its respective mobile phase [56], the recently reported planar Ames bioassay via pH indicator endpoint [15] was performed using the all-in-one 2LabsToGo-Eco [50] to assess the mutagenic potential of coffee by-products, i.e., *Coffea* leaves, cherries, blossoms, and silverskin. The *Salmonella* Typhimurium TA98 strain was used, designed for the detection of frameshift mutations, carrying the hisD3052 mutation, a frameshift within the GCGCGCGC sequence that causes histidine dependence. An *rfa* mutation increases the permeability of its outer membrane, whereas a *uvrB* mutation disables DNA excision repair. Additionally, the presence of the pKM101 plasmid enhances mutagenesis through error-prone repair mechanisms and confers resistance to ampicillin [57].

## 2. Materials and Methods

### 2.1. Chemicals and Materials

Distilled water was obtained from a laboratory tap connected to an in-house water purification system. Methanol, ethanol (96%), 1-propanol, acetone, ethyl acetate, dichloromethane, toluene, *n*-hexane (all chromatography-grade), caffeine (>99%), and 4-nitroquinoline 1-oxide (4NQO, >98%) were purchased from Merck (Darmstadt, Germany). *Salmonella enterica* subspecies *enterica* serotype Typhimurium strain TA98 (*Salmonella* Typhimurium), exposure medium (liquid minimal histidine medium), and *Salmonella* reversion indicator medium (L-histidine-free medium with pH indicator) were delivered by Xenometrix (Allschwil, Switzerland). Chlorogenic acid (CGA, >97%), nutrient broth (for microbiology), glycerol (>99%), ampicillin sodium salt (>99%), potassium hydroxide (≥85%), 2-mL Brand microcentrifuge tubes with 0.3 mm thin caps, and TLC glass plates silica gel 60 F_254_, 10 cm × 10 cm, were purchased from Carl Roth (Karlsruhe, Germany). *Coffea canephora* leaves of the Old Paradenia variety from Badra Estates, Chikmagalur, India, and a *Coffea canephora/arabica* silverskin blend (obtained as a roasting by-product) were provided by the Coffee Store (Mannheim, Germany). *Coffea liberica* cherries (skin and pulp) and blossoms (flowers) from Sarawak, Malaysia, were obtained from Earthlings Coffee Workshop (Kuching, Sarawak, Malaysia).

### 2.2. Extraction of Coffee By-Product Plant Materials

Each finely powdered coffee by-product, i.e., leaves, cherries, silverskin, and blossoms, was accurately weighed (100 mg each), extracted, each in 2 mL of boiling water (ethanol, ethyl acetate, and *n*-hexane were also studied) by ultrasonication for 30 min (35 kHz, 120 W, room temperature; mechanical shaking was also studied), and centrifuged at 3000× *g* for 15 min. The supernatants were ultrafiltrated (0.45 µm polytetrafluoroethylene membrane syringe filter) into 2 mL tubes and stored at 4 °C for further analysis.

### 2.3. Preparation of the Solutions and of the Salmonella Suspension

The 4NQO was dissolved in methanol (1 mg/mL) and applied in increasing amounts of 0.5, 1, 1.5, 2, 2.5, and 3 µg per band. An aqueous ampicillin solution (100 mg/mL) was prepared using sterile distilled water.

For the culture medium, 30 mL of nutrient broth was dispensed into 125 mL Erlenmeyer flasks and sterilized by autoclaving at 120 °C for 20 min. An aqueous ampicillin solution was aseptically added at 25 µg/mL to the cool medium using a 0.2 µm polytetrafluoroethylene membrane syringe filter. For overnight cultivation, 25 µL of a *Salmonella* Typhimurium TA98 cryostock (prepared by harvesting the cell pellet from a 10 mL, 16 h culture, resuspending it in 10 mL of fresh culture medium containing 10% glycerol, and storing it in 0.5 mL cryostock aliquots at −80 °C) was inoculated into 30 mL of culture medium in a 125 mL Erlenmeyer flask and incubated at 37 °C with shaking at 125 rpm in a mini-incubator (Cultura M, 70700R, Almedica, Galmiz, Switzerland) for 16 h. The overnight culture was diluted 1:10 with culture medium to achieve an optical density at 600 nm (OD_600_) of 0.4.

### 2.4. 2LabsToGo-Eco Analysis

The 2LabsToGo-Eco [50] was built from 3D printed parts and components by C.H. during a workshop at Justus Liebig University of Giessen, Giessen, Germany, in September 2024 (www.uni-giessen.de/food, accessed on 28 August 2025). Plant extracts and the reference standards caffeine and CGA (1.5 or 3 µL/band each) were applied 3 times onto the TLC plate, maintaining a distance of 10 mm from the lower, right, and left edges of the plate, with a 4 mm gap between the bands, and dried in a cold stream of air (hair dryer) for 1 min. The 5 mL mobile phase mixture of ethyl acetate–*n*-propanol–water (1:6:3, *v*/*v*/*v*) migrated up to 65 mm. The chromatogram was dried for 4 min and detected under 254 nm ultraviolet (UV) light. Image preprocessing, including negative-peak inversion, signal smoothing, baseline correction, and warping, was performed using the open-source quanTLC software [43]. Densitograms were automatically generated, and automatic peak integration was performed, with the minimum number of increasing and decreasing steps before and after each peak set to one. Method repeatability was evaluated by calculating the absolute and relative standard deviations (SD and *%RSD*) of the peak areas, utilizing spreadsheet software (Microsoft Excel, version 2021, Redmond, WA, USA).

### 2.5. Planar Ames–Vis Bioassay

The mutagenicity bioassay was performed on the chromatogram according to [15]. Each experiment was conducted in four replicates to ensure reproducibility. The chromatogram was adjusted to pH 7.9 by spraying 2 mL of 3% KOH solution, followed by drying in an oven at 120 °C for 15 min. The solvent blank (1.5 µL/band) and the positive control 4NQO (0.5, 1, 1.5, 2, 2.5, and 3 µg/band) were applied above the solvent front, followed by drying for 2 min. A 1.25 mL aliquot of the overnight culture was transferred into a 50 mL centrifuge tube and centrifuged at 3000× *g* for 5 min. The supernatant was discarded, and the resulting cell pellet was resuspended in 2.5 mL of exposure medium and incubated at 37 °C for 40 min. Subsequently, the suspension was centrifuged again at 3000× *g* for 5 min, and the supernatant was removed. The cell pellet was resuspended in 2.6 mL of reversion indicator medium (histidine-free medium containing a pH indicator). The entire suspension was sprayed onto the TLC plate and incubated at 37 °C for 5 h. After the plate was dried for 5 min, detection of mutagens as yellow zones was performed under visible light (Vis).

## 3. Results

### 3.1. Optimization of Extraction and Mobile Phase for TLC Analysis of Coffee By-Products

The extractant solvents were tested based on their polarity, ranging from the nonpolar *n*-hexane, moderately polar ethyl acetate, and more polar ethanol to the most polar hot water. Hot water was the most effective extraction solvent, detecting most compound zones (Supplementary Materials, Table S1). This suggests that most of the compounds in the coffee by-products tested were polar. Water is inexpensive, non-toxic, and nonflammable; however, it may promote bacterial and mold growth during storage [58]. Hot water is the typical medium used for the preparation of cherry, blossom, and leaf infusions intended for consumption. Thus, it best simulated the actual human consumption conditions and exposure. While ethanol extracted polar secondary plant substances including flavonoids, alkaloids, and glycosides, it was avoided due to its limited efficacy in dissolving high-molecular-weight or highly hydrophilic compounds such as polysaccharides, pectins, gums, and waxes [58]. This limitation is particularly relevant for *Coffea* cherry, which is rich in mucilage and polysaccharide constituents [59], often resulting in the precipitation of gel-like materials upon ethanol addition, thereby reducing the extraction efficiency. Ultrasound-assisted extraction uses high-frequency sound waves (>20 kHz) to disrupt plant cell walls, thereby increasing the contact surface area between plant tissues and solvents [56]. However, the comparison of ultrasonication with mechanical shaking revealed no significant difference in the extraction efficiency (Supplementary Materials, Table S1), suggesting that both methods were similarly effective.

Initial tests were conducted by applying the extract solutions with a 2 µL capillary on a silica gel 60 F_254_ TLC plate (cut to smaller formats), followed by development with various solvent combinations (Supplementary Materials, Table S1) in the TLC trough chamber, previously saturated with the mobile phase for 10 min. For mobile phases of lower polarity and thus elution power, most sample components—particularly from the cherry and blossom extracts—remained near the start zone. With increasing mobile phase polarity and thus elution power, compound migration increased. As expected for hot water extracts, polar solvents were best suited, and the ethyl acetate, *n*-propanol, and water (1:6:3, *v*/*v*/*v*) mixture was selected as the mobile phase.

### 3.2. Repeatability of the 2LabsToGo-Eco Analysis

**Figure 1:**
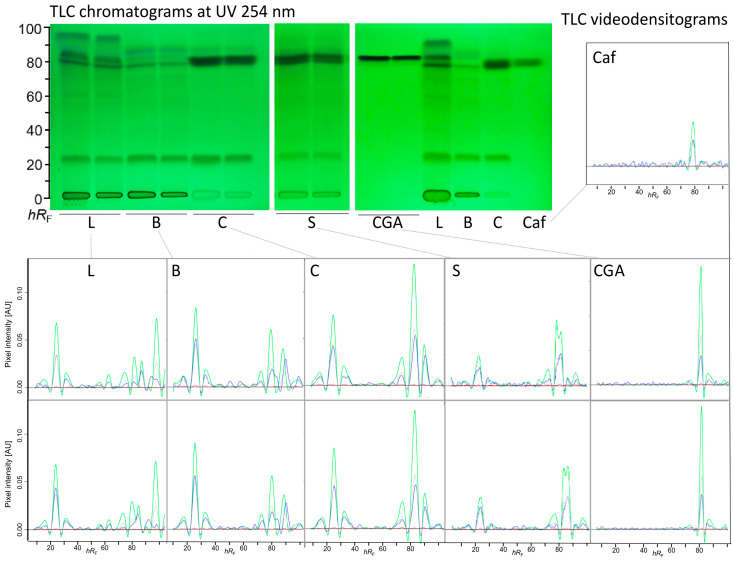
Repeatability of the 2LabsToGo-Eco analysis: TLC chromatograms at UV 254 nm of hot water extracts of leaves (L), blossoms (B), cherries (C), and silverskin (S), each applied in duplicate along with CGA and caffeine (Caf) solutions, 3 µL/band each, separated on a TLC plate silica gel 60 F_254_ with ethyl acetate, *n*-propanol, and water (1:6:3, *v*/*v*/*v*) as well as the corresponding quantTLC videodensitograms with overlaid red/green/blue channels and grey value showing a good method repeatability.

To assess the repeatability of the developed TLC method, the plant extracts were applied in duplicate on the same plate along with the caffeine and CGA reference standards (Figure 1). Both references absorbed at UV 254 nm, and their respective *hR*_F_ values of 82 and 87 matched the compound zones in the sample extracts. Based on the chromatograms at UV 254 nm as well as on the resulting quantTLC videodensitograms, the method exhibited an acceptable visual repeatability.

### 3.3. Reproducibility of the TLC–Ames–Vis Bioautograms

**Figure 2:**
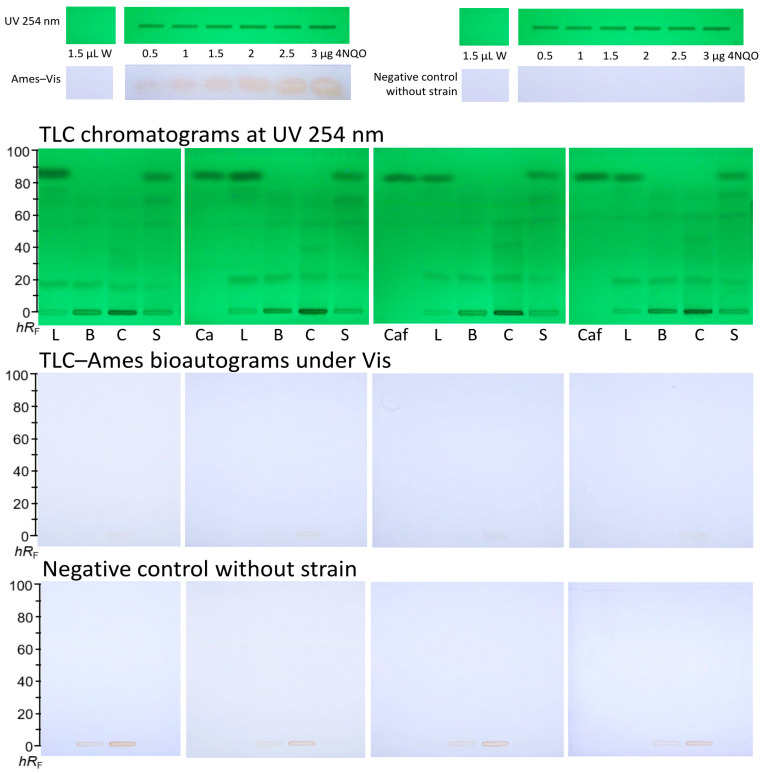
Reproducibility of the TLC chromatograms at UV 254 nm and TLC–planar Ames bioautograms under Vis with *Salmonella* Typhimurium strain TA98 using the 2LabsToGo–Eco: hot water extracts of leaves (L), blossoms (B), cherries (C), and silverskin (S), as well as caffeine solution (Caf), all 1.5 µL/band, analyzed as in Figure 1, along with extractant blank (hot water, W) and positive control 4NQO (0.5–3 µg/band), whereby the yellow mutagenic 4NQO zones verified the proper bioassay performance compared to the negative control without the strain.

The reproducibility of the 2LabsToGo-Eco TLC chromatograms at UV 254 on different plates to confirm the proper sample separation and of the resulting TLC planar Ames bioautograms under Vis using the *Salmonella* Typhimurium TA98 strain was studied (Figure 2). Therefore, after the application of the positive control 4NQO and the extractant blank in the upper plate part, which was not used for the separation, the TLC chromatogram was adjusted to pH 7.9 by spraying a 3% KOH solution. Thereafter, the planar Ames bioassay was successfully performed and verified by the positive control 4NQO, which showed the yellow response indicating mutagenicity. The 4NQO applied at 0.5–3.0 µg/band exhibited a linear dose–response relationship of the yellow response, with peak areas increasing from 4.8 to 8.0 area arbitrary units (AU). Linear regression analysis with a slope of 1.3 AU per amount (in µg) showed a determination coefficient R^2^ = 0.97 (*p* < 0.001), which confirmed the concentration-dependent response. In contrast, the negative control without the strain did not show the yellow response, supporting the specificity and reliability of the planar bioassay.

The tested coffee by-product samples, including hot water extracts of *Coffea* leaves (L), blossoms (B), cherries (C), and silverskin (S), as well as the reference solutions of CGA and caffeine, showed no visible yellow zones in any of the replicates, indicating the absence of any detectable mutagenic activity under the given bioassay conditions. The positive control 4NQO confirmed the proper performance of the assay and its capability to detect mutagenic compounds. The results demonstrated no mutagenic potential of the tested coffee by-products under the given conditions of the planar Ames bioassay with pH indicator substrate with an incubation time of only 5 h and the strain TA98 only. Nevertheless, a longer exposure with an extended incubation time and testing with additional *Salmonella* strains, e.g., TA 100, is recommended to ensure the absence of mutagenicity.

## 4. Discussion

Hot water was the most effective extractant for *Coffea* leaves, blossoms, cherries, and silverskin, detecting most compound zones and indicating that predominantly polar compounds occur in the tested coffee by-products. Coffee cherries, which are naturally rich in pectin [60], formed gel-like particles using ethanol as the extractant. The application of such ethanolic cherry sample extracts with gel-like particles should be avoided using the autosampler of the 2LabsToGo-Eco system. Ethanol should be substituted not only as the extractant, but also as the rinsing solvent between applications, as the interaction between residual ethanol and the pectin-rich matrix may clog the autosampler tubing.

Comprehensive toxicological assessments, including mutagenicity testing, are essential for confirming the safety of coffee by-products intended for human consumption [61,62]. In the in vitro Ames MPF assay [36], *Salmonella* did not grow in the reversion medium, which lacked the essential amino acid histidine. However, *Salmonella* reverted by mutagens did grow and produced acidic metabolites in the reversion medium, which contained the purple bromocresol pH indicator, changing from purple to yellow due to acidification. Non-reverted *Salmonella* did not grow, and the pH indicator remained purple. This principle was used in a recently reported planar Ames bioassay with a pH indicator substrate [15]. However, the incubation was limited to 5 h because of the diffusion of the compound zones with longer incubations. The slightly acidic pH of 6.4 of the original HPTLC plate reduced the detection sensitivity, requiring adjustment to alkaline conditions using 3% KOH before the bioassay performance [15]. The planar Ames bioassay with a pH indicator substrate was successfully performed and verified using the positive control 4NQO and a negative control plate without the strain. The tested coffee by-products, i.e., *Coffea* leaves, blossoms, cherries, and silverskin, did not show mutagenic potential under the given test conditions. Nevertheless, a longer exposure with an extended incubation time and testing with additional *Salmonella* strains, e.g., TA 100, is recommended to confirm their safety for human consumption more comprehensively.

Several biochemical mechanisms could explain the absence of mutagenic activity observed in the coffee by-products tested. Coffee by-products are rich in antioxidant compounds, particularly chlorogenic acids and phenolic compounds [1,2,3,4], which could neutralize reactive intermediates that might otherwise cause DNA damage [63]. The complex mixture of bioactive compounds in coffee by-products may also include natural protective agents that enhance DNA repair mechanisms or upregulate cellular detoxification pathways [64]. Furthermore, in the planar Ames assay, S9 metabolic activation is not integrated, which means that pro-mutagenic compounds will remain undetected. These protective mechanisms and the inherent limitations of the use of a single strain and a short incubation may explain the negative results (absence of mutagenicity) but highlight the importance of complementary testing for a more comprehensive safety assessment.

The 5 h incubation period in the planar bioassay represented a fundamental compromise between detection sensitivity and spatial resolution of zones. While this shortened timeframe reduced sensitivity approximately tenfold compared to standard 48–72 h protocols, it prevented zone diffusion, which would eliminate the advantage of the chromatographic separation. Future method development could explore strategies to extend the incubation while maintaining zone resolution, such as modified TLC plates with reduced diffusion coefficients, gradient temperature profiles to control bacterial growth kinetics, or alternative detection endpoints requiring shorter exposure times. However, the current format should be considered a screening tool for detecting strongly mutagenic compounds rather than a replacement for comprehensive safety assessments.

The observed variability between replicate analyses highlights an important consideration for natural product safety assessment. The variability is probably due to the inherent heterogeneity of botanical matrices, where bioactive compounds may be unevenly distributed within plant tissues, combined with the analytical challenges of working with microvolume applications that can amplify natural compositional differences. Such matrix heterogeneity underscores a fundamental challenge in the safety assessment of natural products and supports the necessity for expanded sample sets encompassing multiple batches, origins, and processing conditions to capture the full spectrum of natural variability over a longer period of time.

Another limitation concerns potential matrix effects from naturally occurring compounds in coffee by-products. Pigments, organic acids, and antioxidants present in these complex matrices could potentially interfere with the pH indicator readout, either masking mutagenic responses or generating false signals. Future investigations could include spiking experiments with known mutagens of coffee by-products to evaluate potential matrix interference and ensure the reliability of negative results with complex botanical matrices.

Despite these limitations, the findings well align with previous studies that have similarly confirmed the safety of coffee by-products [26,27]. In one study, the safety of whole coffee fruits in both powdered and concentrated forms was reported using bacterial mutagenicity and mammalian genotoxicity test systems. Oral toxicity studies at high doses in rats showed good tolerability, with minor, non-adverse effects, such as reduced feed intake and reduced body weight gain, likely due to poor palatability rather than toxicity. No significant clinical, behavioral, or histopathological changes were observed. The established no-observed-adverse-effect level (NOAEL) was 50,000 mg/kg, supporting the safety of coffee fruit extracts in food applications [26]. In another study, a coffee silverskin extract was confirmed to be microbiologically safe, non-cytotoxic, and non-genotoxic based on the HepG2 liver cell line and comet assays. The results showed that the coffee silverskin extract and its primary component, CGA, did not induce DNA damage or oxidative stress at the tested concentrations. It also provided protective effects against benzo[*a*]pyrene-induced oxidative DNA damage, likely due to its strong antioxidant activity. These results support the use of this coffee silverskin extract as a safe, chemoprotective, and potentially valuable food ingredient [27].

## 5. Conclusions

Information regarding the safety of coffee by-products is rarely available. This study provides new safety data for coffee blossoms and leaves, which have been less frequently evaluated so far. Based on the results in the existing literature and of the present study, coffee by-products, including cherry and silverskin, seem not to exhibit severe mutagenicity. According to the EFSA guidelines, in vitro genotoxicity/mutagenicity tests with negative results are generally sufficient, and further in vivo studies are not required. However, considering natural variability, the many pesticides used in coffee production, and the different treatments, as well as the fluctuating supply chains, coffee by-products may highly differ. The increasing interest in utilizing coffee by-products in novel food applications calls for powerful toxicological screening techniques. The planar bioassay technology using the affordable 2LabsToGo-Eco proved to be a good option.

## Data Availability

The original contributions presented in this study are included in the article/Supplementary Material. Further inquiries can be directed to the corresponding author.

## Supplementary Materials

The following supporting information can be downloaded at: https://www.mdpi.com/article/10.3390/toxics13090739/s1, Table S1: Tested mobile-phase systems and respective TLC chromatograms at UV 254 nm of leave extracts obtained using water (1), ethanol (2), *n*-hexane (3), and ethyl acetate (4), blossom extracts obtained using water (5), ethanol (6), *n*-hexane (7), and ethyl acetate (8), and cherry extracts obtained using water (9), ethanol (10), *n*-hexane (11), and ethyl acetate (12).
