# Supplementary material for: Coffee By-Products Studied by the Planar Ames Bioassay with pH Indicator Endpoint Using the 2LabsToGo-Eco
Source: Toxics. 2025 Aug 31;13(9):739. doi: 10.3390/toxics13090739 (PMC12473646; doi:10.3390/toxics13090739)
Supplement: Supplementary file 1 [file toxics-13-00739-s001.zip › toxics-3821958-supplementary.pdf]

# Supporting information

# Coffee By-Products Studied by the Planar Ames Bioassay with pH Indicator Endpoint Using the 2LabsToGo-Eco

**Maryam Monazzah [1,2], Cedric Herrmann [2], Gertrud E. Morlock [3], Jannika Fuchs [2], and Dirk W. Lachenmeier [2]**

[1] Erasmus Programme, Department of Comparative Biomedicine and Food Science, University of Padua, Viale dell'Università 16, 35020 Legnaro, Padua, Italy

[2] Chemisches und Veterinäruntersuchungsamt (CVUA) Karlsruhe, Weissenburger Strasse 3, 76187 Karlsruhe, Germany

[3] Institute of Nutritional Science, Chair of Food Science, Justus Liebig University Giessen, Heinrich-Buff-Ring 26–32, 35392 Giessen, Germany; gertrud.morlock@uni-giessen.de

**Table S1.** Tested mobile phase systems and respective TLC chromatograms at UV 254 nm of leave extracts obtained using water (1), ethanol (2), *n*-hexane (3), and ethyl acetate (4), blossom extracts obtained using water (5), ethanol (6), *n*-hexane (7), and ethyl acetate (8), and cherry extracts obtained using water (9), ethanol (10), *n*-hexane (11), and ethyl acetate (12); manually applied volumes can differ.

| No. | Components | Proportions (*v*/*v*) | Polarity | Extraction method | TLC chromatograms at UV 254 |
|---|---|---|---|---|---|
| 1 | Ethyl acetate<br>Ethanol<br>*n*-Hexane<br>Water | 1/5/1/3 | Moderately polar but contains significant nonpolar content | Shaking | 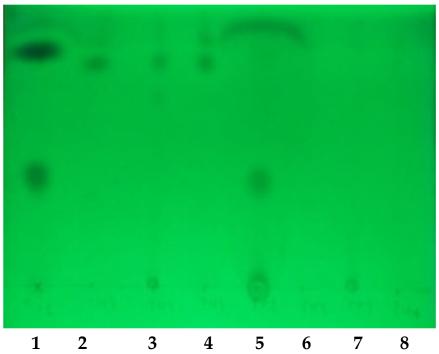  |
| 2 | Ethyl acetate<br>Toluene<br>Ethanol<br>Water | 4/1/0.5/0.5 | Slightly more polar, still contains non-polar toluene | Shaking | 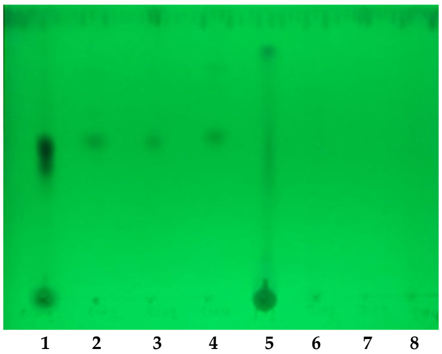  |
| 3 | Toluene<br>Ethyl acetate<br>Formic acid<br>Water | 1/3/0.5/0.3 | More polar due to presence of formic acid and water | Sonication | 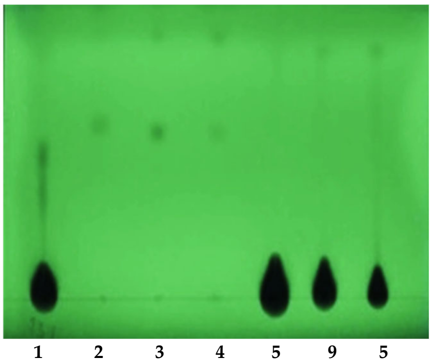  |
| 4 | Ethyl acetate<br>Water | 7/3 | Medium-low polarity | Shaking | 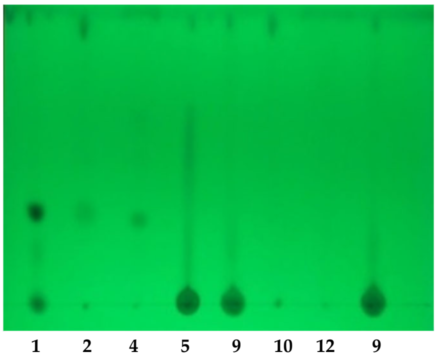  |

| | | | | | |
|---|---|---|---|---|---|
| 5 | Ethyl acetate<br>Ethanol<br>Water | 8/2/0.5 | Mainly ethyl acetate; slightly polar | Shaking | 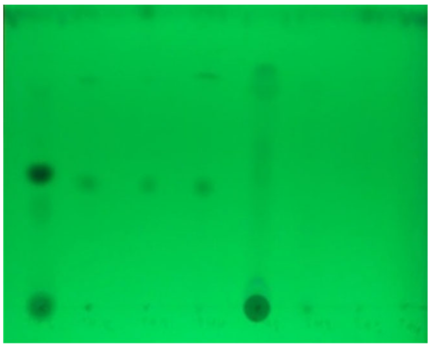<br>1 2 3 4 5 6 7 8 |
| 6 | Ethyl acetate<br>Ethanol<br>Water | 6/3/1 | Moderate polarity; higher ethanol content | Shaking | 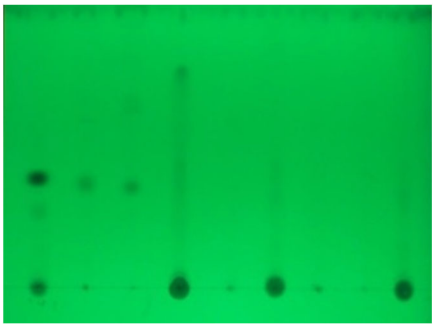<br>1 2 4 1 6 9 10 12 9 |
| 7 | Ethyl acetate<br>Ethanol<br>Water | 5/3/2 | Higher water content; increased polarity | Shaking | 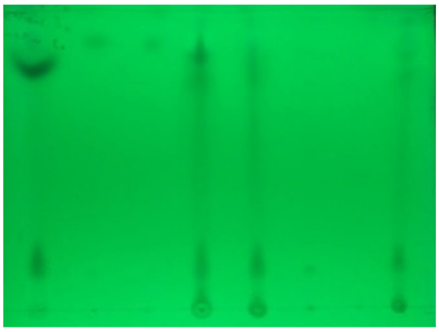<br>1 2 4 5 9 10 12 9 |
| 8 | Ethyl acetate<br>Ethanol<br>Water | 3/4/3 | Significant water and ethanol; more polar | Shaking | 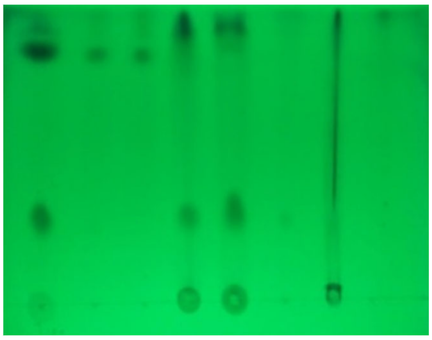<br>1 2 4 5 9 10 12 9 |

| | | | | | |
|---|---|---|---|---|---|
| 9 | Ethyl acetate<br>Methanol<br>Water | 3/4/3 | Slightly more polar due to methanol | Sonication | 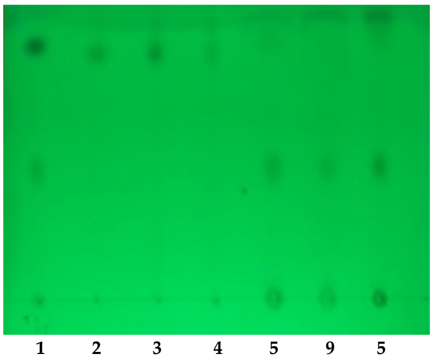 |
| 10 | Ethyl acetate<br>Methanol<br>Water | 7/2/1 | Polarity increased due to methanol | Shaking | 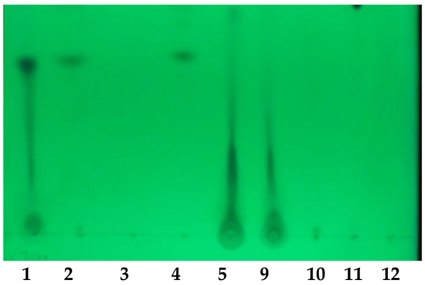 |
| 11 | Ethyl acetate<br>1-Propanol<br>Water<br>Formic acid | 1/1/0.4/0.2 | High polarity from acid and alcohols | Sonication | 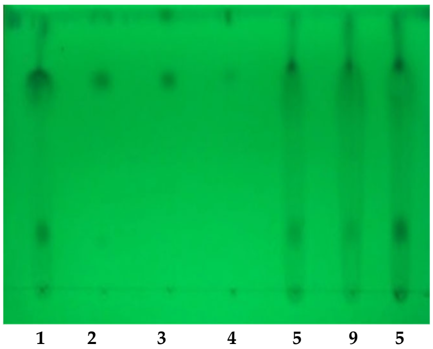 |
| 12 | Ethyl acetate<br>1-Propanol<br>Water | 1/6/3 | Strong polarity due to high n-propanol and water | Shaking | 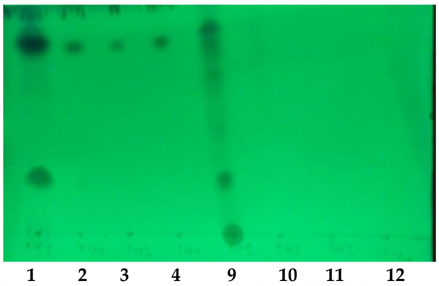 |
| 13 | Ethyl acetate<br>1-Propanol<br>Water | 1/6/3 | Strong polarity due to high n-propanol and water | Shaking | 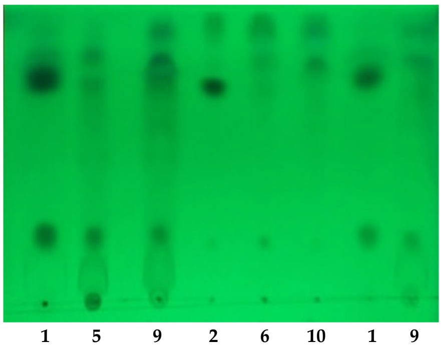 |

| | | | | | |
|---|---|---|---|---|---|
| 14 | *t*-Butanol<br>Propionic acid<br>Water<br>Potassium chloride | 50/12/38<br>+ 0.4 g | Most polar due to salt and strong acid | Shaking | 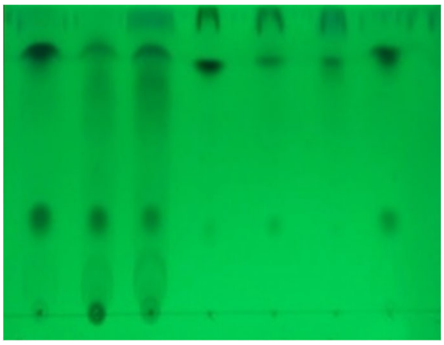<br>1 5 9 2 6 10 1 |
